# Attendance rate and perceived relevance related to type, content, and delivery of current rehabilitation programmes after surgical resection for non-small cell lung cancer

**DOI:** 10.3389/fresc.2024.1447767

**Published:** 2024-12-10

**Authors:** Mette Kaasgaard, Uffe Bodtger, Anders Løkke, Erik Jakobsen, Ole Hilberg

**Affiliations:** ^1^ Pulmonary Research Unit (PLUZ), Department of Medicine, Zealand University Hospital Roskilde and Naestved, Naestved Hospital, Naestved, Denmark; ^2^ Department of Regional Health Research, Faculty of Health Sciences, University of Southern Denmark, Odense, Denmark; ^3^ Department of Medicine, Lillebaelt Hospital, Vejle, Denmark; ^4^ Department of Cardiothoracic and Vascular Surgery, Odense University Hospital, Odense, Denmark; ^5^ Danish Lung Cancer Registry, Odense, Denmark

**Keywords:** rehabilitation, lung cancer, attendance rate, exercise training, quality of life, symptom burden

## Abstract

**Background:**

Surgical resection is the preferred treatment for localised non-small cell lung cancer (NSCLC). Rehabilitation is central in the management of the associated impaired quality of life, high symptom burden, deconditioning, and social-existential vulnerability. Yet, optimal content and delivery of rehabilitation are not yet defined. Therefore, we aimed to investigate the current rehabilitation offers, attendance rate, and perceived relevance related to content or delivery. Moreover, we investigated the current symptom burden in the patients.

**Methods:**

We conducted an observational cohort study in patients who had undergone surgical resection for NSCLC 4–6 months earlier at Odense University Hospital, Denmark. We retrieved demographic data from patient registries, and interviewed patients via telephone concerning availability, uptake, and attendance rate of any rehabilitation offer in their local primary care setting; content and delivery; benefits of attending, experienced relevance and “symptom burden generally” (specially developed questions); and “symptom burden here and now” [Edmonton Symptom Assessment Scale (ESAS)].

**Results:**

We approached 128 patients, reached 115, and interviewed the 100 (87%) patients who consented. In total, 88% (88/100) had received a rehabilitation offer, and 75% (66/88) had participated in programmes that either targeted NSCLC (23%) or were general cancer rehabilitation (33%), pulmonary rehabilitation (12%), online (1%), or other (33%). Disease-specific rehabilitation was significantly related to the highest attendance rate and perception of relevance. High attendance (≥75%) was, moreover, significantly related to the offer being delivered by a physiotherapist and having a focus on physical exercise. General symptoms were physically oriented [dyspnoea (65%), pain (47%), fatigue (78%)] and “mild” in ESAS scoring. No differences were observed in any baseline characteristics.

**Conclusions:**

Rehabilitation after surgical resection for localised NSCLC is delivered heterogeneously in Denmark. Disease-specific rehabilitation was positively related to attendance rate and to the perceived relevance of the offer.

## Introduction

Lung cancer is now the most common cancer worldwide (1), including in Denmark, where the incidence is 5,000 new cases/year, representing 13% of all cancer incidences. At the same time, 5-year survival is increasing, leading to a higher prevalence of long-term survivors (2–6). The main curative treatment for non-small cell lung cancer (NSCLC) is surgical resection of low-stage lung cancer (localised disease with no metastatic spread). Curative treatment may also be achieved using stereotactic radiation therapy (low-stage disease) or concomitant radio-chemotherapy (locally advanced disease) (3, 7).

However, compared to age-matched controls, patients with lung cancer—including the proportion who have undergone surgical resection for NSCLC—have persistently reduced quality of life (QoL), decreased psychological wellbeing, reduced physical and social activity, risk of deconditioning, are marked by symptom clusters (e.g., dyspnoea, cough, pain, fatigue, and dysphonia) and, often, have comorbidities [some of which tobacco related, such as chronic obstructive pulmonary disease (COPD)] (3–5, 8–11).

Besides clinical assessment in a respiratory or oncology outpatient clinic and a computed tomography (CT) scan (7, 10, 12), the recommended follow-up programme includes a relevant rehabilitation offer with, e.g., exercise training, smoking cessation, patient education on self-management to support physical capacity and QoL, and to improve the prognosis (3, 5, 9, 12–15).

However, there is no standardised, lung cancer-specific rehabilitation programme available that addresses the specific needs of the patients after intended curative treatment in lung cancer, including respiratory aspects, QoL, long-term decline in physical capacity, and overall physical and social activity (5, 8, 16), but, as an example, pulmonary rehabilitation (PR) is a multidisciplinary, multi-faceted, and comprehensive intervention based on evidence-based activities (duration: 8–12 weeks), recommended by the European Respiratory Society (ERS) and American Thoracic Society (ATS) to reduce symptoms, increase QoL, and optimise functional capacity (3, 5, 17, 18). A specific core outcome set—specifically for lung cancer rehabilitation—is under development, given a large heterogeneity in outcomes and preferred measures (19) and given that patients with lung cancer are marked by a different and greater symptom burden compared to other cancer types (20).

In Denmark, rehabilitation after a surgical procedure for NSCLC is offered in a municipal primary care setting, but there is a lack of knowledge about the actual availability, uptake, and types of offers, as well as the attendance rate, content and delivery, and perceptions and benefits of the current programmes (10, 16).

### Aims and hypotheses

In this study, we aimed to investigate the availability, uptake, and type of the current rehabilitation offers in primary care settings after a surgical procedure for NSCLC in Denmark, and whether the attendance rate and perceived relevance were related to the type, content, and delivery aspects of the offers. Moreover, we aimed to investigate the needs and symptom burden of the patients 4–6 months after the surgical procedure. We hypothesised that (1) the availability, uptake, and content of the current rehabilitation offer are heterogeneous; (2) the attendance rate and perceived benefits and relevance are related to the type, content, and delivery aspects of the current rehabilitation offer; and (3) patients display a significant symptom burden after their surgical procedure.

## Methods

### Study design and oversight

We conducted an observational study, during which patients were interviewed via telephone between 2 February and 20 March 2024. The study was performed in accordance with the Helsinki II Declaration and obtained all obligatory approvals on 23 November 2023 from the Head Department of Cardiothoracic and Vascular Surgery, Dept. T, Odense University Hospital, the Hospital Director of Lillebaelt Hospital, and the Southern Region of Denmark (J.nr.24/511).

### Participants

We included patients who had undergone surgical resection of stage I or II NSCLC at the Cardiothoracic and Vascular Surgery, Dept. T, Odense University Hospital, Denmark, within the previous year. Patients had been referred for the surgical procedure from the respiratory departments at Zealand University Hospital, Lillebaelt Hospital, and Odense University Hospital. After the surgical procedure, a referral is sent to the patient's municipality which is then obliged to provide a suitable rehabilitation offer.

We aimed to include a total of 100 patients in our study, excluding the proportion who would not consent and who were not reachable across three contact attempts. To ensure that we included patients who had completed any rehabilitation offer after their surgery, we contacted patients, starting with those who had undergone a surgical procedure 4 months before the study.

### Data collection procedure

We retrieved data from the following sources: (1) Demographic and clinical data from the Danish Lung Cancer Registry and electronic medical files, and (2) patient-reported data from telephone-based interviews using a specially developed questionnaire with closed questions (see elaboration under the Outcomes section).

Data from the Danish Lung Cancer Registry were retrieved on 18 January 2024 by the chief physician, EJ, relating to the last 200 patients who had undergone surgery within the last 12 months at the Cardiothoracic and Vascular Surgery, Dept. T, Odense University Hospital. Data were transferred to Professor, DMSc, Head of Research, OH, and variables of interest were entered by two project nurses (specialists in respiratory care at the Respiratory Research Unit, Department of Medicine, Lillebaelt Hospital, Vejle, Denmark) who also retrieved specific data from patient records and collected patient-reported data via telephone.

The overall data collection procedure was developed and defined by the principal investigator, MK, and OH and AL. The specific content of the questionnaire was developed by MK and discussed with OH, EJ, AL, and with the project nurses. The project nurses, moreover, conducted a face-validity procedure in four patients (before the onset of collection of patient-reported data) to ensure that the study scope and questions were found to be relevant, understandable, and sufficiently comprehensive by the patients.

For the interviews, patients were approached by the time at which any rehabilitation offer would recently have been completed, i.e., starting with those operated on 4 months before the study. They received information about the study and their basic rights and were then able to provide their consent to participate in the study.

All data were entered directly into a secure web-based database, SurveyXact, by Ramboll (Rambøll Management Consulting, Aarhus, Denmark). On 25 March 2024, data were imported into statistical software STATA 18 (StataCorp LLC, TX, USA), anonymised, cleaned, and prepared for analysis.

### Outcomes

#### Data from the Danish Lung Cancer Registry

Data from the Danish Lung Cancer Registry comprised baseline demographic data, NSCLC stage, surgery type and classification, any adjuvant treatment, and date of surgical procedure.

#### Data from patient registry

Data from the patient registry comprised data about performance status, body mass index (BMI), and predicted forced expiratory volume in the first second (FEV_1_) percentage, all of which were collected and registered 2–6 weeks prior to the surgical procedure.

#### Patient-reported data collected via telephone

We collected data on self-reported characteristics, using the specially developed questionnaire, and on socio-demographic information (municipality, educational level, employment status, and civil status), smoking status, and self-reported comorbidities and medicine consumption.

Patient-reported data covered the following aspects:

*Availability, uptake, and attendance rate of rehabilitation offer:*

Availability and uptake (e.g., received information about the offer (yes/no), time of information since the surgical procedure, acceptance of the offer (yes/no), and single-choice questions across categories on any reasons behind non-acceptance, attendance rate, or dropout).

*Content and delivery of the offer:*

Content of the rehabilitation offer: (a) “External” characteristics using single-choice questions (setting, type, group-based/individual, and distance to offer); (b) “internal” characteristics using single-choice or multiple-choice questions (duration, frequency, facilitator type, intensity and load, with aspects and elements included).

#### Evaluation of participation—perceived benefits and relevance

1. Perceived benefits from participation: (a) self-experienced (physical fitness, strength, pain, breathing control, dyspnoea, knowledge about disease/body, confidence/hope, symptom burden, and social network); (b) informed by healthcare professional (e.g., physical capacity test or patient-reported questionnaire).
2. Overall evaluation: Experienced satisfaction with the rehabilitation offer, perceived alignments with the patients' needs, perceived relevance, any missing aspects and elements, and perceived appropriateness of the group (if it was a group-based offer).
3. Self-reported evaluation of the level of physical and social activity at present, compared to before the surgical procedure.

*Symptom burden:*

1. Symptoms after surgery, generally: specially developed questions about aspects/symptoms related to respiration, pain, fatigue, vigilance, mood, worry about the future, anxiety, loneliness/isolation, vocal function, and cough [Question: “Have you experienced (…) since the surgical procedure to a degree where it felt prominent to you?” (yes/no)].
2. Symptoms at present, using the “Distress scale,” Edmonton Symptom Assessment System (ESAS), which is a valid and reliable tool to assist in the assessment of nine common symptoms experienced by cancer patients (21). Each symptom's severity *at the time of assessment* is rated from 0 to 10 (0 = symptom absent; 10 = worst possible severity; individual scores in clinical practice: 0 = none, 1–3 = mild, 4–6 = moderate, 7–10 = severe) (21, 22). We calculated sub-scores (23–25): (1) ESAS physical score (six items: pain, fatigue, nausea, drowsiness, appetite, and dyspnoea; scoring range: 0–60), (2) ESAS psychological score (two items: depression and anxiety, scoring range: 0–20), and (3) ESAS total symptom distress score (containing all nine aspects/symptoms, i.e., both physical score, emotional score, and the global item, wellbeing, scoring range: 0–90).

*The needs of patients if* “*non-participant*” *or if* “*no offer*”*:*

1. Engagement in physical activity independently.
2. Extent to which the patient would have liked an offer (to a high degree, to a medium degree, not at all, not certain).

### Analysis

The plan for the analysis was prepared by MK and discussed with OH, AL, and UB. Analyses were performed by MK, using statistical software STATA 18.0 (StataCorp LLC, TX, USA).

#### Descriptive analyses

Initially, we described the overall included study cohort. Continuous data were described as mean ± standard deviation and categorical data were described with the number and percentage. Differences between groups were tested using Student's *t*-test (two-tailed), paired-samples *t*-test, χ^2^, or Fischer's exact test. Statistical significance was reached at *p* < 0.05.

#### Analyses and sub-group analyses

We performed stratified analyses to investigate characteristics in the overall study cohort and sub-groups related to study outcomes and hypotheses:

1. Availability, uptake/acceptance, and content of the offer [stratified into (a) received offer (yes/no), (b) acceptance of the offer (i.e., “non-participant” vs. “participant”].
2. Attendance rate and dropout rate [stratified in “low attendance rate or dropout” (i.e., less than 74% attendance or dropout during the programme) vs. “high attendance rate” (i.e., 75% attendance rate or more) consistent with previous reported rehabilitation attendance rates (26)].
3. Perceived benefits and relevance related to the quality of the offer (i.e., content and delivery).
4. Symptom burden after the surgical procedure and at present.

The STROBE statement checklist for cohort studies was consulted for the reporting of the data.

## Results

### Characteristics

#### Participants

**Figure 1:**
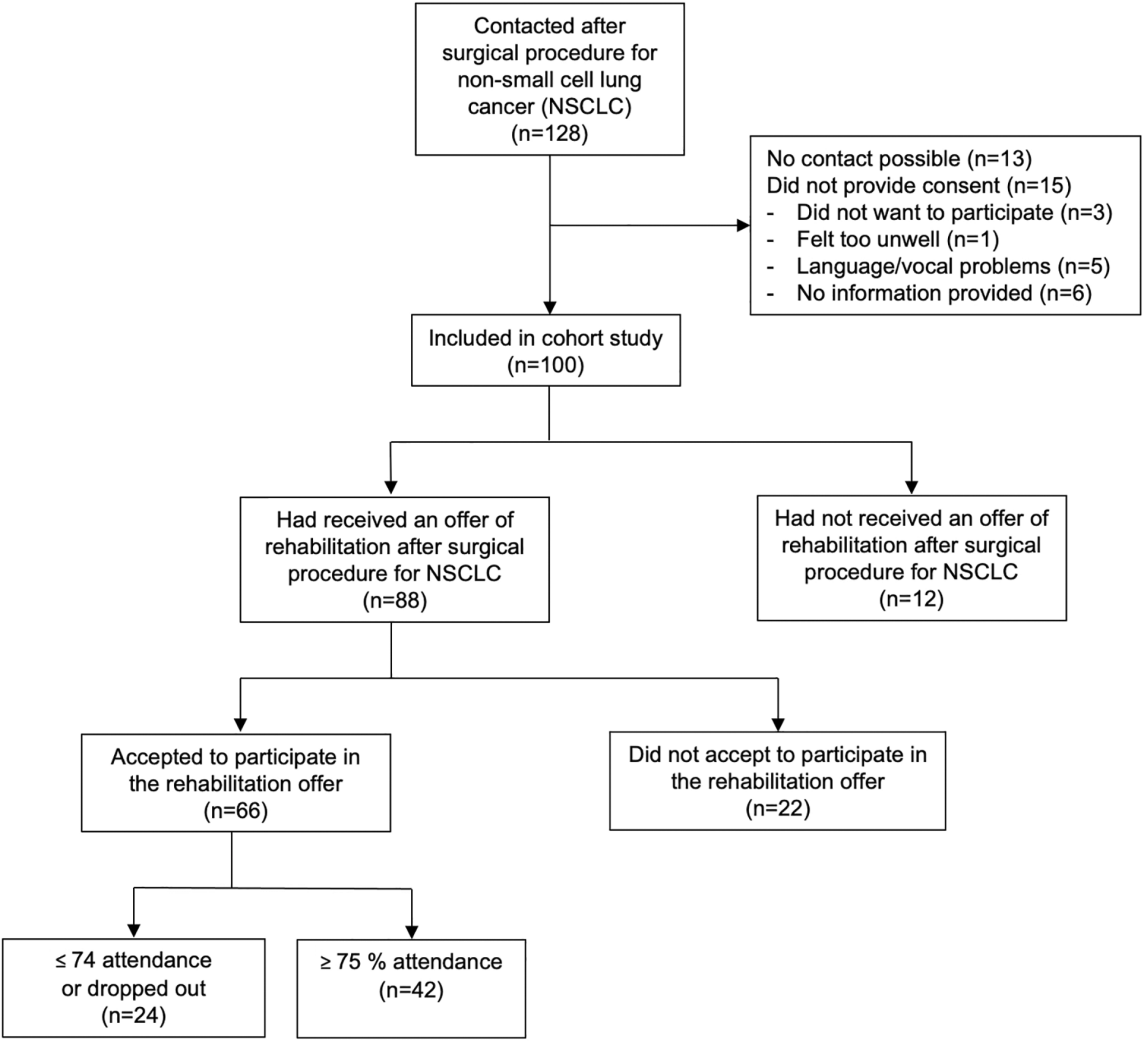
Cohort flow diagram.

The participant flow is depicted in Figure 1. Initially, 128 patients were approached; however, 13 were not reachable. Out of the 115 who were reachable (90%), 100 patients provided consent and were included in the study (87% of 115). The participants represented two Danish regions [Southern Region: *n* = 68 (68%); Zealand Region: *n* = 23 (32%)] and, in total, 35 municipalities (Supplementary Table S1). Across the total cohort included there was an equal distribution between men and women (44% vs. 56%) with a mean age of 72 ± 8; a mean BMI of 27 ± 5, and a mean pre-operative FEV_1_% of 47 ± 20 (Supplementary Table S1).

#### The current rehabilitation offers

Table 1 shows that out of the 88 (88% of 100) who had received an offer of rehabilitation in primary care, 66 (75% of 88) had accepted the offer (i.e., were a participant) (see Figure 1). The vast majority (93%) were contacted by their municipality and most (80%) were contacted within 14 days of the operation.

**Table 1:** Characteristics, symptom burden, and activity in non-participants vs participants.

	*n* = 88		
	*n* = 22	*n* = 66	
	Non-participants	Participants	*p*-value for difference
Characteristics			
Time since surgical procedure (months)	5.2 (2)	5.0 (2)	0.55
Consented to participate in the rehabilitation offer	22 (25%)	66 (75%)	
Age	73 (7)	71 (8)	0.29
Sex, female	12 (55%)	38 (58%)	0.80
BMI	26.4 (6)	27.1 (5)	0.58
FEV1% predicted before surgical procedure	86.3 (24)	87.2 (18)	0.87
Marital status			
Married/cohabiting	15 (68%)	43 (65%)	0.80
Single	7 (32%)	23 (35%)	
Occupational status			
Full or part-time job	2 (9%)	12 (18%)	0.33
Unemployed	0 (0%)	0 (0%)	
Retired	20 (91%)	51 (77%)	
On sick leave	0 (0%)	3 (5%)	
Highest completed education			
Elementary school	3 (14%)	21 (32%)	0.41
Short higher education (under 2 years)	8 (36%)	21 (32%)	
Medium higher education (2–4 years)	9 (41%)	20 (30%)	
Long higher education (5 years or more)	2 (9%)	4 (6%)	
Smoking status			
Current smoker	3 (14%)	7 (11%)	0.94
Former smoker, stopped during the intervention	6 (27%)	15 (23%)	
Former smoker, stopped before the intervention	11 (50%)	37 (56%)	
No, never	2 (9%)	7 (11%)	
Surgical procedure			
Classification of surgical procedure			
Wedge resection	0 (0%)	1 (2%)	0.62
Lobectomy	20 (91%)	62 (94%)	
Segmental resection	2 (9%)	3 (5%)	
Comorbidity or medication usage			
Comorbidity—do you have a doctor's diagnosis of any of these diseases? (multiple answers possible)			
COPD or other lung disease	7 (32%)	23 (35%)	0.80
Cardiovascular disease or kidney disease	11 (50%)	38 (58%)	0.54
Hypertension	13 (59%)	39 (59%)	1.00
Diabetes or other endocrinological diseases	6 (27%)	18 (27%)	1.00
Disease in the musculoskeletal system	14 (64%)	29 (44%)	0.11
Disease of the nervous system	1 (5%)	7 (11%)	0.39
Cancer other than lung cancer	4 (18%)	17 (26%)	0.47
Anxiety or depression	2 (9%)	10 (15%)	0.47
Other disease	3 (14%)	10 (15%)	0.86
Medication usage—do you take medication for any of these illnesses? (multiple answers possible)			
COPD or other lung disease	5 (23%)	20 (30%)	0.50
Cardiovascular disease or kidney disease	10 (45%)	37 (56%)	0.39
Hypertension	13 (59%)	38 (58%)	0.90
Diabetes or other endocrinological diseases	5 (23%)	14 (21%)	0.88
Disease in the musculoskeletal system	11 (50%)	17 (26%)	0.03
Disease of the nervous system	0 (0%)	5 (8%)	0.18
Cancer other than lung cancer	0 (0%)	2 (3%)	0.41
Anxiety or depression	2 (9%)	5 (8%)	0.82
Other disease	1 (5%)	9 (14%)	0.24
Symptom burden			
Symptoms after surgical procedure—have you experienced prominent problems? (yes) (multiple answers possible)			
Breathing-related symptoms/dyspnoea	13 (59%)	47 (71%)	0.29
Pain	13 (59%)	29 (44%)	0.22
Fatigue	16 (73%)	55 (83%)	0.28
Stiffness/tightness in the chest	2 (9%)	22 (33%)	0.03
Notice my body's signals all the time	16 (73%)	51 (77%)	0.66
Concerns about the future	8 (36%)	28 (42%)	0.62
Depressive symptoms	7 (32%)	24 (36%)	0.70
Feelings of anxiety and restlessness	4 (18%)	21 (32%)	0.22
Feeling of being alone/lonely/isolated	4 (18%)	8 (12%)	0.47
Voice problems	6 (27%)	17 (26%)	0.89
Edmonton Symptom Assessment System (ESAS)			
Total ESAS scores			
ESAS physical score (1–6)	7.9 (8)	9.7 (9)	0.40
ESAS psychological score (7–8)	1.0 (3)	1.6 (3)	0.41
ESAS total symptom distress score (1–6 + 7–8 + 9)	10.5 (12)	13.0 (12)	0.40
Individual ESAS scores			
Physical domain			
1. Pain	1.4 (2)	1.4 (2)	0.98
2. Fatigue	1.9 (2)	3.1 (3)	0.07
3. Nausea	0.0 (0)	0.5 (1)	0.15
4. Drowsiness	1.0 (2)	1.0 (2)	0.98
5. Appetite	1.5 (2)	1.3 (3)	0.84
6. Breathlessness	2.0 (3)	2.4 (3)	0.58
Psychological domain			
7. Depression	0.7 (2)	0.9 (2)	0.67
8. Anxiety	0.3 (1)	0.8 (2)	0.31
9. Wellbeing	1.6 (2)	1.7 (2)	0.91
Self-reported change in overall activity compared to before surgical procedure			
Are you currently more or less physically active than before your surgery?			
Less active	7 (32%)	35 (53%)	0.06
Same as before	14 (64%)	23 (35%)	
More active	1 (5%)	8 (12%)	
Are you currently more or less socially active than before your surgery?			
Less active	6 (27%)	15 (23%)	0.48
Same as before	16 (73%)	47 (71%)	
More active	0 (0%)	4 (6%)	

As depicted in Table 2, the majority of the participants (*n* = 66) started rehab within a month after surgery (71%). Most often, the rehabilitation offer was delivered in a community-based healthcare centre (86%). For 30 (46%) participants, the programme lasted 6–12 weeks but was longer for 29 (45%). A frequency of “more than once a week” was reported by 75% and “once a week” by 14%. The type of rehabilitation offer varied between a special offer that targeted lung cancer (23%), mixed cancer rehabilitation (33%), pulmonary rehabilitation (12%), online (2%), or other (including at home with instructions from a physiotherapist, with other diseases, or not specified) (33%). We observed no overall differences between non-participants and participants (Tables 2, 3).

**Table 2:** Characteristics of the rehabilitation offers provided to participants.

	*n* = 66
	Participants
Information and type	
When were you contacted by your municipality?	
Within 7 days	19 (29%)
7–14 days	37 (56%)
More than 14 days	9 (14%)
Do not know	1 (2%)
By whom were you contacted? (two answered “do not know”)	
Municipal healthcare centre (primary care setting)	62 (94%)
Hospital	1 (2%)
Other (e.g., at home or in the facilities of the Danish Cancer Society)	2 (3%)
Which type of rehabilitation offer?	
Special offer for lung cancer	15 (23%)
General cancer rehabilitation	22 (33%)
Pulmonary rehabilitation	8 (12%)
Online	1 (2%)
Other (at home with a physiotherapist, other rehabilitation across diseases, not specified)	22 (33%)
How long after the surgical procedure did the rehabilitation offer start?	
Within 1 month	47 (71%)
1–2 months after OP	10 (16%)
2–3 months after OP	6 (9%)
Do not know	3 (5%)
Where did the rehabilitation offer take place?	
Municipal healthcare centre (primary care setting)	57 (86%)
Hospital	1 (2%)
In the facilities of the Danish Cancer Society	4 (6%)
At home with a physiotherapist	7 (11%)
Online/telemedicine	1 (2%)
Other (private physio)	5 (8%)
Duration, delivery, and intensity	
How many weeks did the rehabilitation offer last? (missing one respondent)	
Under 6 weeks	6 (9%)
6–12 weeks	30 (46%)
Longer time	29 (45%)
How often did the training take place during the course? (missing two respondents)	
Several times a week	48 (75%)
Once a week	9 (14%)
Once every second week	1 (2%)
Do not know	6 (9%)
Was the training delivered in a group or individually?	
In a group	39 (59%)
Individually	28 (42%)
Which things were included in the training?	
Fitness/cardio training	53 (80%)
Strength training	51 (77%)
Body awareness/mental training/mindfulness	7 (11%)
Exercises/training for breathing	33 (50%)
Other (nature, daily life, self-training)	5 (8%)
Which trainer was responsible for the physical training?	
Physiotherapist	61 (92%)
Occupational therapist	2 (3%)
Nurse	9 (14%)
Other (instruction in self-training)	1 (3%)
How hard do you think the physical training was? *N* = 63 (missing three respondents)	
Too hard	3 (5%)
Adequate	50 (79%)
Too easy	4 (6%)
Do not know	6 (10%)
Patient education	
Did to rehabilitation offer contain any other components besides physical training? *N* = 63 (missing three respondents)	
Yes	26 (41%)
If so, which ones? (multiple answers possible)	
Smoking cessation	10 (15%)
Knowledge about my disease	14 (21%)
Breathing techniques	9 (14%)
Nutrition/diet	21 (32%)
Managing symptoms	15 (23%)
Dealing with everyday life	12 (18%)
Techniques for dealing with anxiety/worry	11 (17%)
Other (psychologist, psychotherapist, sexuality, wilderness activity, choir singing)	9 (14%)
Attendance rate and dropout	
Participation—how much of the course did you attend overall?	
The entire course	35 (53%)
3/4 of the course	7 (11%)
1/2 of the course	2 (3%)
1/4 of the course	4 (6%)
Less than 1/4 of the course	8 (12%)
Course interrupted, but agreed to resume later	6 (10%)
Dropout	5 (8%)
Reasons for dropout during the course (multiple answers possible) (% out of 66)	
Too sick or ill to participate	12 (18%)
Did not want to participate	2 (3%)
Did not feel like I matched with the group	2 (3%)
Prevented due to participation in other treatment	10 (16%)
Other reason (COVID-19, vacation, personal reasons, pain, transportation, timing, work)	18 (27%)
Reasons for dropout during the course (multiple answers possible) (% out of 66)	
Too sick to participate	2 (3%)
Did not want to participate	0 (0%)
Didn't find the activity/offer relevant	5 (8%)
Didn't feel like I fit in the group	3 (5%)
Thought the training was too hard	2 (3%)
Was hindered due to participation in other treatment	1 (3%)
Other reason (trained at home, work, programme started too soon, bad timing)	8 (12%)

**Table 3:** Benefits and evaluation of the offer provided to participants.

	*n* = 66
	Participants
Self-reported benefits	
Do you feel that you have benefited from participating? (missing four respondents)	
Yes	47 (76%)
No	10 (16%)
Do not know	5 (8%)
Which improvements have you experienced from participating? (multiple answers possible)	
Improved fitness	36 (54%)
Increased physical strength	32 (48%)
Less pain	2 (3%)
Less breathlessness	17 (26%)
Better control over my breathing	19 (29%)
Knowledge about my body, my disease, and my symptoms	5 (8%)
More courage to face everything	20 (30%)
Social community/being with like-minded people	8 (12%)
Overall, have had fewer symptoms	7 (11%)
Other (become happier, can walk longer, more motivated)	4 (6%)
Have not experienced any improvements	4 (6%)
Tests and response from healthcare professional	
Were tests/examinations performed during the course? (multiple answers possible)	
Yes, walking test or other physical test	19 (29%)
Yes, questionnaire	1 (2%)
Yes, other [attendance, spirometry, conversations, sit-to-stand (STS) test, repetitions]	7 (11%)
No	36 (55%)
Were you informed by a healthcare professional whether you had improved?	
Yes	24 (41%)
No	31 (53%)
Do not know	4 (7%)
If yes, which aspects had improved? (multiple answers possible)	
Walking distance	15 (23%)
Quality of life	5 (8%)
Other [general, repetitions, control over breathing, STS test, machines]	11 (17%)
Had not improved	4 (6%)
Do not know	3 (5%)
Was not informed	11 (17%)
Evaluation of group-based or individually based offer	
If you participated in a group, to what extent did you feel that you matched well with the group? (% out of 39)	
To a high degree	24 (62%)
In between	7 (18%)
Not at all	7 (18%)
Do not know	1 (3%)
If you did not feel you matched with the group, what was the primary reason? (multiple answers possible)	
The others were much older than me	4 (10%)
The others were much younger than me	0 (0%)
The others were in better shape than me	0 (0%)
The others were in worse shape than me	4 (10%)
Our challenges/issues were too different	7 (18%)
Other (not as sick as others, training at too low a level, got more sick/feeling bad from seeing the others who were more sick than me)	4 (10%)
If you participated in individual training (e.g., at home), how satisfied were you with the offer? (% out of 28)	
To a high degree	21 (75%)
In between	3 (11%)
Not at all	1 (4%)
Do not know	3 (11%)
Overall evaluation,% of 66	
To what extent do you feel that the offer has been relevant to you overall?	
To a high degree	46 (77%)
In between	8 (13%)
Not at all	6 (10%)
Is there anything that you missed in the course? (multiple answers possible)	
Physical training	5 (8%)
Training in breathing techniques	5 (8%)
Education about my disease	4 (6%)
Guidance in daily life	4 (6%)
Psychological/mental aspects	9 (14%)
Smoking cessation	0 (0%)
Nutrition/diet	5 (8%)
Other (consideration of symptoms, more training, evaluation/test, adjusted level)	7 (11%)
Nothing—was satisfied with the course as it was	40 (61%)
Are you currently more or less physically active than before your surgery?	
Less active	35 (53%)
Same as before	23 (35%)
More active	8 (12%)
Are you currently more or less socially active than before your surgery?	
Less active	15 (23%)
Same as before	47 (71%)
More active	4 (6%)

### Primary study outcome

#### Attendance rate related to type, content, and delivery, to perceived benefits, and to relevance of the rehabilitation offer

Table 4 shows that a high attendance rate was related to the type, content, and delivery of the rehabilitation offer. Among those with high attendance, a larger proportion was offered a disease-specific offer (high: 31% vs. low or dropout: 8%; *p* = 0.04), the offer was delivered more often delivered by a physiotherapist (98% vs. 83%; *p* = 0.04), and the offer more often comprised physical exercise components more often (fitness: 88% vs. 67%; *p* = 0.04, strength: 86% vs. 62%; *p* = 0.03).

**Table 4:** Benefits and evaluation of the offer related to low vs high attendance.

	*n* = 66		
	*n* = 24	*n* = 42	
	≤74% attendance or dropout	≥75% attendance	*p*-value for difference
Characteristics of offer			
Which type of rehabilitation offer?			
Special offer for lung cancer	2 (8%)	13 (31%)	0.04
General cancer rehabilitation	8 (33%)	14 (33%)	1.00
Pulmonary rehabilitation	3 (12%)	5 (12%)	0.94
Online	0 (0%)	0 (0%)	1.00
Other (at home with physiotherapist, other training across diseases, not specified)	11 (46%)	11 (26%)	0.10
Which things were included in the training?			
Cardio training	16 (67%)	37 (88%)	0.04
Strength training	15 (62%)	36 (86%)	0.03
Body awareness/mental training/mindfulness	2 (8%)	5 (12%)	0.65
Exercises/training for breathing/respiration	9 (38%)	24 (57%)	0.12
Other (nature, daily life, self-training)	2 (8%)	3 (7%)	0.86
Which trainer was responsible for the physical training?			
Physiotherapist	20 (83%)	41 (98%)	0.04
Occupational therapist	0 (0%)	2 (5%)	0.28
Nurse	3 (12%)	6 (14%)	0.84
Other (instruction in self-training)	1 (4%)	1 (2%)	0.68
How hard do you think the physical training was?			
Too hard	3 (14%)	0 (0%)	<0.001
Just right	10 (48%)	40 (95%)	
Not hard enough	3 (14%)	1 (2%)	
Do not know	5 (24%)	1 (2%)	
Evaluation of group-based or individual-based offers			
Did the training take place in a group or individually?			
In a group	12 (50%)	27 (64%)	0.26
Individually	9 (38%)	19 (45%)	0.54
Do not know	3 (12%)	0 (0%)	0.02
If you participated in a group, to what extent did you feel you matched well with the group? (% out of 39)			
To a high degree	5 (13%)	19 (49%)	0.23
In between	2 (5%)	5 (13%)	
Not at all	4 (10%)	3 (8%)	
Do not know	1 (3%)	0 (0%)	
If you did not feel you matched with the group, what was the primary reason? (multiple answers possible)			
The others were much older than me	2 (8%)	2 (5%)	0.56
The others were much younger than me	0 (0%)	0 (0%)	
The others were in better shape than me	0 (0%)	0 (0%)	
The others were in worse shape than me	3 (12%)	1 (2%)	0.09
Our challenges/issues were too different	5 (21%)	2 (5%)	0.04
Other (not as sick as others, the training was too low level, got more sick/feeling bad from seeing the others who were more sick than me)	4 (17%)	0 (0%)	0.01
If you participated in individual training (e.g., at home), how satisfied were you with the offer? (% out of 28)			
To a high degree	5 (18%)	16 (57%)	0.01
In between	1 (4%)	2 (7%)	
Not at all	1 (4%)	0 (0%)	
Do not know	4 (14%)	0 (0%)	
Self-reported benefits			
Do you feel that you have benefited from participating?			
Yes	10 (50%)	37 (88%)	<0.01
No	7 (35%)	3 (7%)	
Do not know	7 (35%)	2 (5%)	
Which improvements have you experienced from participating? (multiple answers possible)			
Gotten in better shape	9 (38%)	27 (64%)	0.04
Increased physical strength	8 (33%)	24 (57%)	0.06
Less pain	2 (8%)	0 (0%)	0.06
Less breathlessness	1 (4%)	16 (38%)	<0.01
Better control over my breathing	2 (8%)	17 (40%)	<0.01
Knowledge about my body, my disease, and my symptoms	1 (4%)	4 (10%)	0.43
More courage to face everything	4 (17%)	16 (38%)	0.07
Social community/being with like-minded people	0 (0%)	8 (19%)	0.02
Overall, have had fewer symptoms	2 (8%)	5 (12%)	0.65
Other (become happier, can walk longer, more motivated)	1 (4%)	3 (7%)	0.63
Have not experienced any improvements	2 (8%)	2 (5%)	0.56
Tests and response from healthcare professional			
Were tests/examinations performed during the course? (multiple answers possible)			
Yes, walking test or other physical test	2 (8%)	17 (40%)	<0.01
Yes, questionnaire	0 (0%)	1 (2%)	0.45
Yes, other (attendance, spirometry, conversations, sit-to-stand (STS) test, repetitions)	1 (4%)	6 (14%)	0.20
No	16 (67%)	20 (48%)	0.13
Were you informed by a healthcare professional whether you had improved?			
Yes	3 (18%)	21 (50%)	0.01
No	14 (82%)	17 (40%)	
Do not know	0 (0%)	4 (10%)	
If yes, which aspects had improved? (% out of 66) (multiple answers possible)			
Walking distance	2 (8%)	13 (31%)	0.04
Quality of life	0 (0%)	5 (12%)	0.08
Other [general, repetitions, control over breathing, STS test, training machines]	1 (4%)	10 (24%)	0.04
Had not improved	1 (4%)	3 (7%)	0.63
Do not know	2 (8%)	1 (2%)	0.26
Was not informed	5 (21%)	6 (14%)	0.49
Overall evaluation			
To what extent do you feel the offer has overall been relevant to you?			
To a high degree	9 (50%)	37 (88%)	<0.001
In between	3 (17%)	5 (12%)	
Not at all	6 (33%)	0 (0%)	
Is there anything that you missed in the course? (multiple answers possible)			
Physical training	4 (17%)	1 (2%)	0.04
Training in breathing techniques	4 (17%)	1 (2%)	0.04
Education about my disease	3 (12%)	1 (2%)	0.09
Guidance in daily life	1 (4%)	3 (7%)	0.63
Psychological/mental aspects	4 (17%)	5 (12%)	0.59
Smoking cessation	0 (0%)	0 (0%)	
Nutrition/diet	3 (12%)	2 (5%)	0.25
Other (consideration of symptoms, more training, evaluation/test, adjusted level)	2 (8%)	5 (12%)	0.65
Nothing—was satisfied with the course as it was	10 (42%)	30 (71%)	0.02

In addition, a high attendance rate was related to perceived benefits (88% vs. 50%; *p* ≤ 0.01), including improved fitness (64% vs. 38%; *p* = 0.04), less dyspnoea (38% vs. 4%; *p* ≤ 0.01), enhanced breathing control (40% vs. 8%; *p* ≤ 0.01), and positive socialising with peers (19% vs. 0%; *p* = 0.02) (Table 4). A larger proportion of those with a high attendance rate had performed a walking distance test compared to those with a low attendance rate or who had dropped out (40% vs. 8%; *p* ≤ 0.01). Furthermore, a greater proportion of those with a high attendance rate had been informed by a health professional about any improvements (50% vs. 18%; *p* = 0.01) and had improved their walking distance during the programme (31% vs. 8%; *p* = 0.04).

There was no difference between group-based and individual-based delivery (*p* = 0.23), but fewer of those with a low attendance rate or who had dropped out found that they matched well with the other participants in their group (*p* = 0.04; *p* = 0.01) and fewer were content with individual-based delivery (18% vs. 57%; *p* = 0.01). Furthermore, fewer of those with a low attendance rate or who had dropped out found the intensity and load appropriate (48% vs. 95%; *p* ≤ 0.001), fewer were satisfied with the offer (18% vs. 57%; *p* = 0.02), and fewer found the offer relevant (50% vs. 88%; *p* ≤ 0.001).

We observed no differences in characteristics between those with a high attendance rate [42 (64%)] and those with a low attendance rate or who had dropped out [24 (36%)] (Supplementary Table S2).

### Secondary outcomes

#### Symptom burden of patients in general and at present

In the overall cohort (*n* = 100) (Supplementary Table S1), the most dominant symptoms that participants reported having experienced since the surgical procedure were physically oriented [dyspnoea (65%), pain (47%), fatigue (78%), vigilance (74%)], psychologically oriented [worry about the future (40%), mood/depression (35%), anxiety (29%)], and vocal problems (27%).

At present, however, no individual item in the ESAS tool was above “mild” in all the analyses, reflecting experienced symptoms “here and now” (Supplementary Table S1 and Tables 1–4). We observed no overall differences in ESAS scoring between non-participants and participants (Table 1) or between those with a high attendance rate and those with a low attendance rate or who dropped out (Table 4).

#### Self-reported level of physical and social activity compared to before the surgical procedure

In the overall population (*n* = 100), 48% reported being less physically active compared to before the surgical procedure, whereas 42% were equally active compared to before, and only 10% were more active. Regarding social activity, 25% reported being less socially active, 71% were equally active compared to before, and only 4% were more active. We observed no overall differences between non-participants and participants (Tables 2, 3), or between high attendance and low attendance or dropout (Table 4).

#### The needs of patients if they were a “non-participant” or if there was no offer

In non-participants and those who did not receive an offer (*n* = 33), 93% reported having been physically active by themselves more than once a week (88%) since the operation, however, mainly with light intensity and load (91%) (Supplementary Table S2). In those who did not receive an offer (*n* = 12), one (8%) would have liked an offer, two (17%) were indifferent, six (50%) would not have liked an offer, and three (25%) declared themselves to be uncertain. The majority had had a lobectomy procedure (9/12; 75%) (Supplementary Table S4).

## Discussion

In this observational study, we found that attendance rate and perceived benefits and relevance were related to the type, content, and delivery of the current rehabilitation offers, which were delivered in primary care settings after surgical resection for localised NSCLC.

The majority of study participants were invited to participate in rehabilitation (88%) (Table 1), which is in line with the current guidelines (16). It remains unclear why 12 (8% out of 100) apparently reported not having received an invitation for a rehabilitation offer. As was hypothesised, the rehabilitation type and focus were heterogeneous in our study, and only less than one in four received disease-targeted (lung cancer) rehabilitation (Table 2). However, it has previously been addressed that the needs of patients with lung cancer are different from other cancer types, which means that only a disease-specific rehabilitation offer will encompass all relevant aspects (10, 19, 20). Another aspect was the content and delivery of the offers, and we found that a relevant means to define a “high-quality” offer would be whether the offers were disease-specific, comprised physical exercise components, were delivered by physiotherapists, and included measurements and evaluations of the patient’s progress since the programme baseline. In line with our hypotheses, a high attendance rate was related to each of these components (Table 4). At the same time, a high attendance rate was related to the perceived benefits related to physical components, respiratory components, psychosocial aspects, and to whether the participants had found the programme relevant, suitable, and satisfactory. As adherence level and dropout remain persistent challenges (8, 18, 27, 28), these findings support the need to establish disease-specific and “high-quality” rehabilitation programmes with perceived relevant content and outcomes for this particular patient group (10, 19, 20). Exercise training is the gold standard activity across all rehabilitation programmes (5, 18, 27, 29), although a 2018 systematic review by Sommer et al. found only “low quality” evidence regarding exercise capacity and the physical component of HRQoL (5). In contrast, a Cochrane review found “high-quality” evidence regarding exercise capacity, suggested improvements in the physical component of HRQoL, and, moreover, suggested decreased dyspnoea (although with “very low quality” evidence) (15).

Well-known barriers in rehabilitation include lack of motivation and ability to perform physical exercise training. Nevertheless, exercise training is highly significant in improving or preserving physical activity and functional capacity (30), although a 360° approach is needed as, for example, emotional aspects are also important to consider (20). In our study, we found that common reasons for declining to participate included feeling that the rehabilitation offer was not suitable and that the participants could not identify with the other participants in the programme (Supplementary Table S2). Notably, a larger proportion of those with a low attendance rate or who dropped out specifically reported that they did not feel that they matched with the other participants in the group, compared to those with a high attendance rate (Table 4). Therefore, we greatly anticipate the results of the international Delphi consensus study to guide preferred outcomes and measures to further develop and evaluate future disease-specific high-quality rehabilitation programmes for people with lung cancer (19).

In addition, we encourage the investigation of other non-pharmacological and evidence-based activities as supplements or alternatives to exercise training for those who cannot or will not perform exercise training, both within the course of a rehabilitation programme and after the programme. A patient-centred approach may stimulate engagement in relevant and motivational activities and may address both physiological and psychosocial aspects (18, 19, 28, 31). As an example, we recently demonstrated that singing, delivered as a structured training modality, conferred measurable improvements in physical capacity and quality of life equally to physical exercise training and in a dose-response manner within a 10 weeks' community-based pulmonary rehabilitation programme for people with COPD (26, 32). Furthermore, singing may be related to improved respiratory muscle strength and coordination and to improved dyspnoea control (33). Interestingly, an editorial in The European Respiratory Journal suggested that engagement in an activity such as singing may build confidence to participate in exercise training at a later point, either within a rehabilitation programme or independently (34). To further investigate the potential of singing as a structured training modality, we are currently planning a multicentre randomised controlled trial on the effects of singing on exercise capacity, QoL, and symptom burden, specifically for people with NSCLC.

To define the optimal content and delivery of a future disease-specific rehabilitation offer, it is essential to gain further knowledge about the current symptom burden of the patients. In this study, participants reported that they had had multiple symptoms early after the surgical procedure (Table 1 and Supplementary Tables S1, S2), which is in keeping with previous studies in which especially physically oriented symptoms were observed (10, 11). However, we only observed “mild” symptoms from the ESAS questionnaire (21, 22). One explanation for this discrepancy could be that the ESAS questionnaire specifically asks about the symptoms “here and now,” which may influence the participants’ interpretation of the questions. Another explanation could be that lung cancer resection is increasingly often performed as less invasive video- or robot-assisted thoracoscopic surgery, which is associated with a lower symptom burden and better long-term outcomes than open thoracotomy (11, 35), leading to lower participation in rehabilitation and healthy responder selection bias (36). Nevertheless, a less time-specific tool to evaluate symptom burden would likely have to be more relevant, such as the EORTC 30-item Quality of Life Questionnaire (QLQ-C30) (37, 38), which has also been previously been applied in a Danish context and which demonstrated that a proportion of patients had long-term symptoms after a surgical procedure for NSCLC (11).

Notably, a pivotal aspect in the management of lung cancer is smoking cessation (10, 39). In the present study, 15% were currently smokers and 23% had stopped smoking during the programme (Supplementary Table S1). Nevertheless, only 15% reported that smoking cessation was included in the rehabilitation programme (Table 2). This underlines the need to include smoking cessation in future rehabilitation programmes to support smoking cessation and to prevent patients from taking up smoking again (10).

### Strengths and limitations

Overall, the present study provides important knowledge for future research and for the further development of relevant and motivating rehabilitation programmes to support patients after a surgical procedure for NSCLC.

Our study has several strengths: First, to our knowledge, this is the first study to investigate the availability, uptake, attendance rate, dropout, and the type, content, and delivery of current rehabilitation offers in a real-life study. Second, the study is based on patient-reported data regarding content and perceived benefits and relevance, reflecting actual patient perspectives outside the scope of research and guidelines. Third, we included important perspectives about the symptom burden after the surgical procedure. Fourth, we included a large proportion of study participants and, overall, found consistency in our findings and with the existing literature. This may all contribute to the generalisability and credibility of the study.

The study also has several limitations. First, it was an observational study and the inclusion of patient perspectives regarding availability, uptake, attendance rate, dropout, and regarding type, content, and delivery may lead to recall bias. Second, the inclusion of only self-reported and subjective data may lead to a risk of design and recall bias. Third, the quality of reporting solely relies on patient-reported perceptions and interpretations and may not correspond to the perspectives and evaluations of health professionals.

## Conclusion

Rehabilitation after surgical resection for non-small cell lung cancer is delivered heterogeneously in Denmark with less than 25% receiving a disease-specific rehabilitation offer. Nevertheless, a disease-specific and high-quality rehabilitation programme was positively related to a high attendance rate, to reported benefits, and to perceived relevance of the offer.

## Data Availability

The raw data supporting the conclusions of this article will be made available by the authors, without undue reservation.
