# Supplementary material for: Attendance rate and perceived relevance related to type, content, and delivery of current rehabilitation programmes after surgical resection for non-small cell lung cancer
Source: Front Rehabil Sci. 2024 Dec 10;5:1447767. doi: 10.3389/fresc.2024.1447767 (PMC11666536; doi:10.3389/fresc.2024.1447767)
Supplement: Supplementary file 1 [file Datasheet1.pdf]

Frontiers in Rehabilitation Sciences: Pulmonary Rehabilitation (Special Issue: Psychosocial Issues and Interventions in Pulmonary Rehabilitation)

Supplementary files for manuscript:

Attendance rate and perceived relevance related to type, content, and delivery of current rehabilitation programmes after surgical resection for non-small cell lung cancer

Authors
Mette Kaasgaard, PhD (1,2); Uffe Bodtger, PhD (1,2); Anders Løkke, DMSc (1,3); Erik Jakobsen, MPM (4,5); Ole Hilberg, DMSc (1,3).

Overview of supplementary tables:

Page 2: Supplementary Table S1: Characteristics, symptom burden, and activity in the study cohort (n=100)

Page 5: Supplementary Table S2: Characteristics, symptom burden, and activity - low vs high attendance

Page 7: Supplementary Table S3: Those who were offered rehabilitation after surgical procedure, but declined it (n=22 (88-66))

Page 7: Supplementary Table S4: Those who did not receive a rehabilitation offer after surgical procedure (n=12)

SUPPLEMENARY TABLE S1

Characteristics, symptom burden, and activity in the study cohort (n=100)

	n=100<br>Complete study cohort
**Characteristics**	
**Regions**	
**Region of Southern Denmark**	
Number of participants	68 (68%)
Number of municipalities (% out of 36)	22 (61%)
**Region Zealand**	
Antal deltagere	32 (32%)
Number of municipalities (% out of 36)	14 (39%)
**Have you recieived a rehabilitation offer after surgical procedure, yes**	88 (88%)
**Age**	71.3 (7.9)
**Sex, Female**	56 (56%)
**BMI**	26.6 (5.2)
**FEV1 % predicted before surgical procedure**	46.5 (19.5)
**Marital status**	
Married/cohabiting	65 (65%)
Single	35 (35%)
**Occupational status**	
Full or part-time job	14 (14%)
Unemployed	0 (0%)
Retired	83 (83%)
On sick leave	3 (3%)
**Highest completed education**	
Elementary school	31 (31%)
Short higher education (under 2 years)	31 (31%)
Medium higher education(2-4 years)	32 (32%)
Long higher education (5 years or more)	6 (6%)
**Smoking status**	
Yes	15 (15%)
No, stopped during the intervention	23 (23%)
No, stopped before the intervention	53 (53%)
No, never	9 (9%)
**Surgical procedure**	
**Performance-status after surgical procedure (missing information: 25)**	
mean value	
0	47 (47%)
1	25 (25%)
2	4 (4%)
3	0 (0%)
4	0 (0%)
**Neoadjuvant treatment (missing information: 48)**	
Yes	16 (16%)
No	36 (36%)
**Type of surgical procedure**	
Thoracoscopy	62 (62%)
Thoracotomy	38 (38%)
**Classification of surgical procedure**	
Wedge resection	3 (3%)
Lobectomy	91 (91%)
Segmental resection	6 (6%)
**T-classification (cancer)**	

T1a	4 (4%)
T1b	27 (27%)
T1c	28 (28%)
T2a	22 (22%)
T2b	3 (3%)
T3	16 (16%)
**N-classification**	
N0	87 (87%)
N0/3	1 (1%)
N1	12 (12%)
**M-classification**	
MO	100 (100%)
**Comorbidity and medication usage**	
**Comorbidity - do you have a doctor's diagnose of any of these diseases? (multiple answers possible)**	
COPD or other lung disease	35 (35%)
Cardiovascular disease or kidney disease	56 (56%)
Hypertension	58 (58%)
Diabetes or other endocrinological diseases	26 (26%)
Disease in the musculoskeletal system	50 (50%)
Disease of the nervous system	9 (9%)
Cancer other than lung cancer	25 (25%)
Anxiety or depression	14 (14%)
Other disease	14 (14%)
**Medication usage - do you take medication for any of these illnesses? (multiple answers possible)**	
COPD or other lung disease	28 (28%)
Cardiovascular disease or kidney disease	54 (54%)
Hypertension	56 (56%)
Diabetes or other endocrinological diseases	20 (20%)
Disease in the musculoskeletal system	31 (31%)
Disease of the nervous system	5 (5%)
Cancer other than lung cancer	2 (2%)
Anxiety or depression	8 (8%)
Other disease	11 (11%)
**Symptom burden**	
Breathing-related symptoms/dyspnoea	65 (65%)
Pains	47 (47%)
Fatigue	78 (78%)
Stiffness/tightness in the chest	29 (29%)
Notice my body's signals all the time	74 (74%)
Concerns about the future	40 (40%)
Depressive symptoms	35 (35%)
Feelings of anxiety and restlessness	29 (29%)
Feeling of being alone/lonely/isolated	13 (13%)
Voice problems	27 (27%)
**Edmonton Symptom Assessment System (ESAS)**	
**Total ESAS scores**	
ESAS physical score (1-6)	8.9 (8.6)
ESAS psychological score (7-8)	1.6 (3.3)
ESAS total symptom distress score (1-6 + 7-8 + 9)	12.1 (12.1)
**Individual ESAS scores**	
Physical domain	
1: Pain	1.3 (2.4)
2: Fatigue	2.7 (2.7)
3: Nausea	0.3 (1.2)
4: Drowsiness	0.9 (1.9)
5: Appetite	1.3 (2.5)

6: Breathlessness	2.1 (2.5)
Psychological domain	
7: Depression	0.8 (1.7)
8: Anxiety	0.7 (1.9)
9: Wellbeing	1.5 (2.2)
**Self-reported change in overall activity compared to before surgical procedure**	
**Are you currently more or less physically active than before your surgery?**	
Less active	48 (48%)
Same as before	42 (42%)
More active	10 (10%)
**Are you currently more or less socially active than before your surgery?**	
Less active	25 (25%)
Same as before	71 (71%)
More active	4 (4%)

SUPPLEMENTARY TABLE S2

Characteristics, symptom burden, and activity - low vs high attendance			
	n=66		
	n=24	n=42	
	≤74% attendance or drop out	≥75% attendance	p-value for difference
**Characteristics**			
**Time since surgical procedure (months)**	5.2 (1.8)	5.3 (2.8)	0.85
**Age**	68.2 (8.6)	72.0 (7.5)	0.06
**Sex**			
Male	11 (46%)	17 (40%)	0.67
Female	13 (54%)	25 (60%)	
**BMI**	26.8 (5.0)	27.3 (5.4)	0.72
**FEV1 % predicted before surgical procedure**	87.1 (18.5)	87.2 (18.7)	0.98
**Marital status**			
Married/cohabiting	14 (58%)	29 (69%)	0.38
Single	10 (42%)	13 (31%)	
**Work situation**			
In work	5 (21%)	7 (17%)	0.91
Unoccupied	0 (0%)	0 (0%)	
Pensioner	18 (75%)	33 (79%)	
On sick leave	1 (4%)	2 (5%)	
**Highest completed education**			
Elementary school	4 (17%)	17 (40%)	0.08
Short higher education (under 2 years)	12 (50%)	9 (21%)	
Medium higher education(2-4 years)	7 (29%)	13 (31%)	
Long higher education (5 years or more)	1 (4%)	3 (7%)	
**Smoking status**			
Yes	2 (8%)	5 (12%)	0.78
No, stopped during the intervention	7 (29%)	8 (19%)	
No, stopped before the intervention	13 (54%)	24 (57%)	
No, never	2 (8%)	5 (12%)	
**Comorbidity og medicine use**			
**Comorbidity - do you have a doctor's diagnose of any of these diseases? (multiple answers possible)**			
COPD or other lung disease	10 (42%)	13 (31%)	0.38
Cardiovascular disease or kidney disease	13 (54%)	25 (60%)	0.67
Hypertension	12 (50%)	27 (64%)	0.26
Diabetes or other endocrinological diseases	6 (25%)	12 (29%)	0.75
Disease in the musculoskeletal system	14 (58%)	15 (36%)	0.08
Disease of the nervous system	3 (12%)	4 (10%)	0.71
Cancer other than lung cancer	7 (29%)	10 (24%)	0.63
Anxiety or depression	3 (12%)	7 (17%)	0.65
Other disease	6 (25%)	4 (10%)	0.09
**Medication use - do you take medication for any of these illnesses? (multiple answers possible)**			
COPD or other lung disease	9 (38%)	11 (26%)	0.34
Cardiovascular disease or kidney disease	13 (54%)	24 (57%)	0.81
Hypertension	12 (50%)	26 (62%)	0.35
Diabetes or other endocrinological diseases	4 (17%)	10 (24%)	0.49
Disease in the musculoskeletal system	8 (33%)	9 (21%)	0.29
Disease of the nervous system	3 (12%)	2 (5%)	0.25
Cancer other than lung cancer	1 (4%)	1 (2%)	0.68
Anxiety or depression	2 (8%)	3 (7%)	0.86
Other disease	5 (21%)	4 (10%)	0.20

**Symptom burden**			
**Symptoms after surgical preocedure - have you experienced prominent problems? (yes) (multiple answers possible)**			
Difficulty breathing/shortness of breath	15 (62%)	32 (76%)	0.24
Pains	13 (54%)	16 (38%)	0.21
Fatigue	17 (71%)	38 (90%)	0.04
Stiffness/tightness in the chest	9 (38%)	13 (31%)	0.59
Notice my body's signals all the time	19 (79%)	32 (76%)	0.78
Concerns about the future	10 (42%)	18 (43%)	0.93
Bad mood/depression	7 (29%)	17 (40%)	0.36
Feelings of anxiety and restlessness	5 (21%)	16 (38%)	0.15
Feeling of being alone/lonely/isolated	4 (17%)	4 (10%)	0.39
Voice problems	3 (12%)	14 (33%)	0.06
**Edmonton Symptom Assessment System (ESAS)**			
**Total ESAS scores**			
ESAS physical score (1-6)	11.2 (9.4)	8.9 (8.3)	0.30
ESAS psychological score (7-8)	1.0 (2.4)	2.0 (3.6)	0.24
ESAS total symptom distress score (1-6 + 7-8 + 9)	13.8 (11.7)	12.6 (12.0)	0.70
**Individual ESAS scores**			
Physical domain			
1: Pain	1.8 (2.6)	1.2 (2.2)	0.34
2: Fatigue	3.9 (2.9)	2.6 (2.5)	0.06
3: Nausea	1.0 (2.2)	0.2 (0.6)	0.03
4: Drowsiness	0.7 (1.5)	1.2 (2.4)	0.30
5: Appetite	1.5 (2.6)	1.2 (2.4)	0.63
6: Breathlessness	2.4 (2.8)	2.5 (2.9)	0.92
Psychological domain			
7: Depression	0.8 (1.8)	0.9 (1.9)	0.70
8: Anxiety	0.2 (0.8)	1.0 (2.2)	0.10
9: Wellbeing	1.5 (2.3)	1.7 (2.3)	0.77
**Self-reported change in overall activity compared to before surgical procedure**			
**Are you currently more or less physically active than before your surgery?**			
Less active	15 (62%)	20 (48%)	0.27
Same as before	8 (33%)	15 (36%)	
More active	1 (4%)	7 (17%)	
**Are you currently more or less socially active than before your surgery?**			
Less active	8 (33%)	7 (17%)	0.29
Same as before	15 (62%)	32 (76%)	
More active	1 (4%)	3 (7%)	

SUPPLEMENTARY TABLE S3

Those who were offered rehab after surgical procedure, but declined it (88-66=22)	
	n=22
**What was the primary reason why you declined to participate? (multiple answers possible)**	
Did not have the energy	4 (18%)
Was too sick	3 (14%)
Too far to the training location	2 (9%)
Did not have the time	3 (14%)
Did not receive an offer	2 (9%)
Other	18 (82%)
-Physiotherapist assessed it as not relevant	
-Sitting together with others who received chemo…	
-Preferred to handle it myself	
-Wanted to train together with spouse	
-Did not feel like it	
-Did not fit in with the others	
-Received the offer too late	

SUPPLEMENTARY TABLE S4

Those who did not receive a rehabilitation offer after surgical procedure (n=12)	
	n=12
**To what extent would you wish that you had received an offer?**	
To a high degree	1 (8%)
In between	2 (17%)
Not at all	6 (50%)
Do not know	3 (25%)
**Surgical procedure**	
**Classification of surgical procedure**	
Wedge resection	2 (17%)
Lobectomy	9 (75%)
Segmental resection	1 (8%)
